# Non‐coding RNA involvement in the pathogenesis of diabetic cardiomyopathy

**DOI:** 10.1111/jcmm.14510

**Published:** 2019-06-26

**Authors:** Wei Zhang, Weiting Xu, Yu Feng, Xiang Zhou

**Affiliations:** ^1^ Department of Cardiology The Second Affiliated Hospital of Soochow University Suzhou China; ^2^ Department of Endocrinology The Second Affiliated Hospital of Soochow University Suzhou China

**Keywords:** diabetic cardiomyopathy, non‐coding RNA, pathogenesis

## Abstract

In recent years, the incidence of diabetes has been increasing rapidly, which seriously endangers human health. Diabetic cardiomyopathy, an important cardiovascular complication of diabetes, is characterized by myocardial fibrosis, ventricular remodelling and cardiac dysfunction. It has been documented that mitochondrial dysfunction, oxidative stress, inflammatory response, autophagy, apoptosis, diabetic microangiopathy and myocardial fibrosis are implicated in the pathogenesis of diabetic cardiomyopathy. With the development of molecular biology technology, accumulating evidence demonstrates that non‐coding RNAs (ncRNAs) are critically involved in the molecular mechanisms of diabetic cardiomyopathy. In this review, we summarize the pathological roles of three types of ncRNAs (microRNA, long ncRNA and circular RNA) in the progression of diabetic cardiomyopathy, which may provide valuable insights into the pathogenesis of diabetic cardiovascular complications.

## 1 INTRODUCTION

Diabetic cardiomyopathy is a type of cardiac dysfunction that develops in the absence of hypertensive heart disease, coronary artery disease and valvular heart disease.^1^ It is characterized by myocardial fibrosis, ventricular enlargement and cardiac dysfunction that ultimately leads to heart failure. Emerging evidence implicates that mitochondrial dysfunction, oxidative stress, inflammatory response, autophagy, apoptosis, diabetic microangiopathy and myocardial metabolic abnormalities are involved in the development of diabetic cardiomyopathy.^2^ Hyperglycaemia can exert adverse effects on myocardial tissue through various mechanisms, including metabolic disturbance, microvascular impairment and subcellular structure abnormalities.

**Figure 1:**
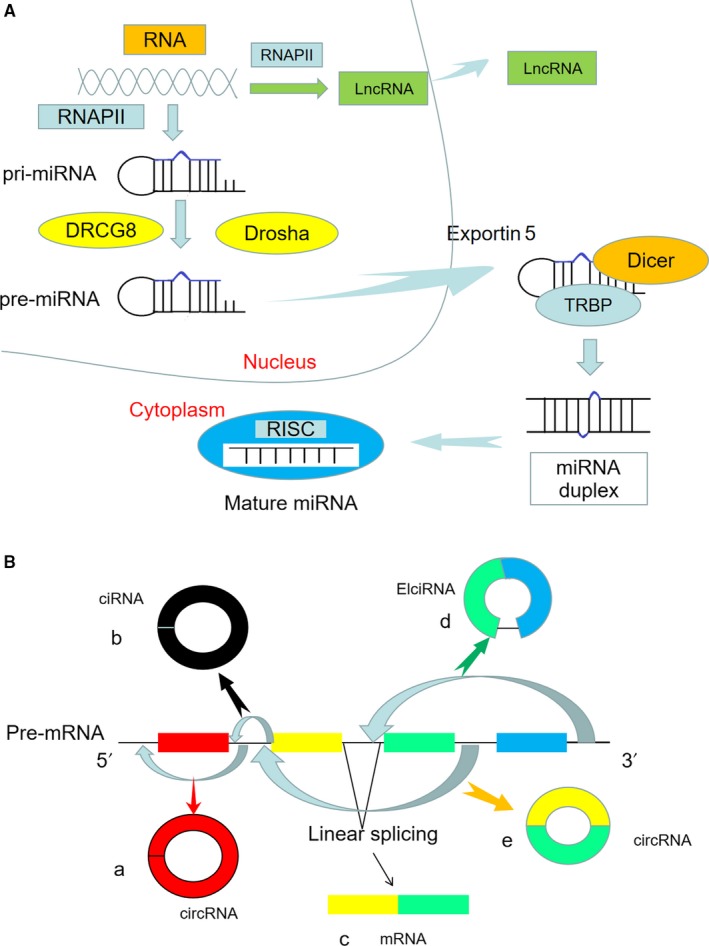
A, The biogenesis of miRNA and lncRNA. The miRNA is transcribed as primary miRNA (pri‐miRNA) by RNA polymerase II (RNAPII). Following processing by the Drosha and DRCG8, precursor miRNA (pre‐miRNA) is exported from the nucleus by exportin 5. Then, it undergoes further processing by Dicer and TAR RNA‐binding protein (TRBP) to generate mature miRNA loaded into the RNA‐induced silencing complex (RISC). The lncRNA is transcribed mostly by RNAPII, and its biogenesis process is similar to miRNA. B, The biogenesis of circRNA. a, e. circRNA by direct back‐splicing; b. circular intronic RNA (ciRNA) by back‐splicing; c. mRNA by canonical splicing; d. exon‐intron circRNA (EIciRNA) by lariat‐driven circularization

In recent years, non‐coding RNAs (ncRNAs) have important functional implications for human health and disease.^3^ There are many types of ncRNAs,^4^ and the main classes of functional ncRNAs that are not translated into proteins include microRNA (miRNA), long ncRNA (lncRNA) and circular RNA (circRNA). The biogenesis of ncRNAs is complex, and the specific process is shown in Figure 1. It has been reported that ncRNAs participate in the pathogenesis of multiple cardiovascular diseases by both transcriptional and post‐transcriptional regulation.^5^, ^6^, ^7^ In the present review, we summarize the important roles of miRNA, lncRNA and circRNA in the pathogenesis of diabetic cardiomyopathy.

## 2 miRNAs

miRNAs are highly conserved and single‐stranded ncRNAs that include 20‐22 nucleotides. Their primary function is to negatively modulate gene expression through binding to the target mRNA and subsequently inducing its degradation or suppressing the translation.^8^ miRNAs can regulate the translation of more than 60% of protein‐coding genes. The available evidence indicates that miRNAs can regulate cardiac hypertrophy,^9^ myocardial fibrosis,^10^ oxidative stress and apoptosis,^11^ mitochondrial dysfunction,^12^ epigenetic modification,^13^ cardiac electrical remodelling 14 and other pathophysiological changes,^15^ which are associated with diabetic cardiomyopathy.

### 2.1 Cardiac hypertrophy

Multiple miRNAs have been reported to modulate cardiac hypertrophy in diabetic cardiomyopathy. Antihypertrophic miRNAs include miR‐1,^16^ miR‐30c,^17^ miR‐181a,^18^ miR‐150,^9^ miR‐133a 19 and miR‐373.^20^ Prohypertrophic miRNAs include miR‐208a,^21^ miR‐451^22^ and miR‐195.^23^ Raut et al^18^ indicated that miR‐30c and miR‐181a could synergistically regulate p53‐p21 pathway in cardiac hypertrophy induced by diabetes. Feng et al^19^ reported that miR‐133a was downregulated in hypertrophic cardiac tissue under high glucose conditions and miR‐133a overexpression prevented hypertrophic changes in cardiomyocytes. Ikeda et al^16^ showed that miR‐1 attenuated cardiomyocyte hypertrophy by negative regulation of calcium signalling components calmodulin, Gata4 and Mef2a. The miR‐1/ mitochondrial calcium uniporter (MCU) axis is involved in the dynamic adaptation of cardiomyocytes to hypertrophy.^24^ Involvement of miR‐1 in cardiomyocyte development and hypertrophic remodelling is indicated by the inverse correlation of MCU expression with muscle‐specific miR‐1. miR‐150 can inhibit cardiomyocyte hypertrophy induced by high glucose through targeting the transcriptional coactivator p300.^9^ In addition, miR‐208a was found to promote cardiac hypertrophy by inhibiting myostatin and GATA4 expression and upregulating β‐myosin heavy chain.^21^

### 2.2 Cardiomyocyte apoptosis, autophagy and pyroptosis

Some miRNAs have been identified to modulate cardiomyocyte apoptosis, autophagy and pyroptosis, which are responsible for the pathogenesis of diabetic cardiomyopathy. Increased expression of miR‐1,^25^ miR‐30b,^26^ miR‐206,^27^ miR‐144,^11^ miR‐195,^23^ miR‐208a,^28^ miR‐320,^29^ miR‐378,^30^ miR‐483‐3p^31^ and miR‐34a^32^ can promote apoptosis in diabetic cardiomyopathy. In addition, miR‐30c,^33^ miR‐221,^34^ miR‐30a, miR‐133a and miR‐212^35^ are associated with autophagy regulation in the diabetic heart. Qiao et al^31^ indicated that miR‐483‐3p overexpression promoted cardiomyocyte apoptosis in diabetic mice by suppressing the expression of insulin‐like growth factor‐1 (IGF‐1). Zheng et al^23^ revealed that silencing of miR‐195 could inhibit myocardial hypertrophy and improve cardiac function in diabetes by reducing cardiomyocytes apoptosis and promoting angiogenesis in cardiac endothelial cells. Shan et al^27^ showed that miR‐1 and miR‐206 post‐transcriptionally modulated Hsp60 expression, which consequently resulted in cardiomyocyte apoptosis induced by high glucose. Recently, miR‐30c overexpression was found to inhibit BECN1 induction and the subsequent autophagy in diabetic myocardium and improve cardiac structure and function in diabetic mice.^33^ Su et al^34^ revealed that miR‐221 could inhibit autophagy and promote heart failure by modulating p27/CDK2/mTOR pathway. Pyroptosis is a pro‐inflammatory programmed cell death and plays important roles in the pathogenesis of diabetic cardiomyopathy. Li et al^15^ reported that miR‐30d could promote cardiomyocyte pyroptosis in diabetic cardiomyopathy by regulating foxo3a. Moreover, miR‐9 was found to reduce cardiomyocyte pyroptosis induced by hyperglycaemia through targeting ELAV‐like protein 1.^36^

### 2.3 Myocardial fibrosis

Myocardial fibrosis is a typical pathological characteristic of diabetic cardiomyopathy, and it is regulated by miR‐133a,^10^ miR‐15a/b,^37^ miR‐21,^38^ miR‐29^39^ and miR‐200b.^40^ Liu et al^38^ revealed that miR‐21 was upregulated in cardiac fibroblasts exposed to high glucose and could accelerate collagen synthesis through the c‐Jun N‐terminal kinase and p38 signalling pathways. miR‐15a/b were found to be downregulated in the myocardium of diabetic patients and consequently activate fibrotic signalling of transforming growth factor‐β receptor‐1 and connective tissue growth factor (CTGF).^37^ In addition, van Rooij et al^39^ showed that the miR‐29 family targeted a series of mRNAs encoding proteins such as multiple collagens, fibrillins and elastin, which are involved in the process of fibrosis. Recently, Feng et al^40^ demonstrated that miR‐200b mediated endothelial‐to‐mesenchymal transition in diabetic mice and contributed to increased myocardial fibrosis in diabetic cardiomyopathy.

### 2.4 Oxidative stress

Oxidative stress is critically involved in the pathogenesis of diabetic cardiomyopathy. It has been documented that miR‐1,^41^ miR‐22,^42^ miR‐144,^11^ miR‐195,^23^ miR‐200c^43^ and miR‐503^44^ are involved in the regulation of hyperglycaemia‐induced oxidative stress. Yildirim et al^41^ indicated that miR‐1 expression in cardiomyocytes was decreased under high glucose treatment, and overexpression of miR‐1 protected against diabetes‐induced cardiac oxidative damage. Through in vivo and in vitro experiments, Tang et al^42^ found that enforced expression of miR‐22 could attenuate oxidative injury by upregulating Sirt 1 in diabetic cardiomyopathy. Zhang et al^43^ showed that miR‐200c increased COX‐2 expression in endothelial cells by suppressing ZEB1 expression and promoting prostaglandin E2 production, thus reducing endothelium‐dependent relaxation. Furthermore, Miao et al^44^ demonstrated that miR‐503 expression was upregulated in diabetic cardiomyopathy, and miR‐503 participated in the protective effects of Phase II Enzyme Inducer CPDT by regulating nuclear factor erythroid 2‐related factor 2/antioxidant response elements, which is the critical antioxidant signalling pathway in the body and can regulate the gene expression of several antioxidative enzymes.^45^

### 2.5 Other pathophysiological processes

miRNAs can also actively participate in the pathogenesis of cardiac structural damage,^46^ mitochondrial dysfunction,^47^ inflammatory response,^48^, ^49^ angiogenic regulation^50^ and myocardial electrical remodelling.^14^ Arnold et al^46^ reported that miR‐29 overexpression in a diabetes model was associated with cardiac structural damage and accompanied by decreased expression of myeloid cell leukaemia 1, a protein that promotes cell survival. In another diabetes model, increased miR‐141 expression affected ATP production by decreasing mitochondrial phosphate transport.^47^ Reddy et al^48^ indicated that disruption of the negative regulatory loop involving miR‐200 and Zeb1 increased inflammatory response in vascular smooth muscle cells under diabetic conditions. In the diabetic heart, miR‐146a was associated with elevated inflammatory factor and extracellular matrix protein production and cardiac functional alterations.^49^ In addition, miR‐193‐5p was found to be actively involved in the development of diabetic cardiomyopathy, possibly through negatively regulating its downstream gene IGF2.^50^ Panguluri et al^14^ suggested that miR‐301a mediated regulation of voltage‐gated potassium channel Kv4.2 and participated in the electrical remodelling in diabetic cardiomyopathy.

Taken together, a number of miRNAs have been identified to be involved in the pathogenesis of diabetic cardiomyopathy via different signalling pathways. Future research should focus on the interaction of miRNAs with the regulatory network, which may help to further understand the molecular mechanisms of diabetic cardiomyopathy. In addition, some circulating miRNAs have the potential to be used as biomarkers in the diagnosis and prognosis of diabetic cardiovascular complications.^51^, ^52^ Furthermore, the current research on miRNAs may provide valuable insight into the future treatment of diabetic cardiomyopathy.

## 3 lncRNAs

lncRNAs, a class of transcripts which are longer than 200 nucleotides without protein‐coding potential, have been implicated in multiple biological processes, including genomic imprinting, transcriptional regulation, nuclear organization and compartmentalization, RNA splicing and nuclear‐cytoplasmic trafficking.^53^, ^54^, ^55^, ^56^ In recent years, growing evidence has suggested that lncRNAs can actively participate in the pathogenesis of diverse cardiovascular diseases, including diabetic cardiomyopathy.^57^

### 3.1 Myocardial fibrosis

Myocardial fibrosis is an important pathological change in diabetic cardiomyopathy. Zhang et al^58^ reported that lncRNA‐AK081284 expression was increased in cardiac fibroblasts exposed to high glucose, while IL‐17 knockdown abrogated the upregulation of AK081284 induced by high glucose. In addition, AK081284 overexpression was found to promote the production of collagen and transforming growth factor β1 (TGFβ1) in cardiac fibroblasts. Thus, the IL‐17/AK081284/TGFβ1 pathway is involved in collagen production induced by high glucose. Thomas et al^59^ showed that lncRNA‐ANRIL regulated structural and functional abnormalities in the diabetic hearts by regulating the expression of extracellular matrix (ECM) protein and vascular endothelial growth factor (VEGF). These alterations modulated by ANRIL might be mediated by epigenetic modifier p300 and polycomb repressive complex 2 complex. Tao et al^60^ suggested that H19 negatively modulated DUSP5 expression in cardiac fibroblast and fibrosis tissues. H19 was found to promote cardiac fibroblast proliferation via inhibition of DUSP5/ERK1/2 axis. In addition, a recent study by Piccoli et al^61^ demonstrated that silencing of lncRNA‐Meg3 could inhibit the production of matrix metalloproteinase‐2 (MMP‐2), leading to reduced myocardial fibrosis and improved cardiac dysfunction.

The competing endogenous RNA (ceRNA) theory has been proposed that protein‐coding RNAs and ncRNAs can communicate with each other to modulate gene expression by competing for binding to shared miRNAs.^62^ Tao et al^63^ suggested that lncRNA‐GAS5 could function as a ceRNA to regulate PTEN/MMP‐2 signalling pathway by sponging miR‐21, thus playing a suppressive role in cardiac fibrosis. Liang et al^64^ revealed that lncRNA‐PFL contributed to cardiac fibrosis through promoting fibroblast‐myofibroblast transition via competitively binding to let‐7d.

### 3.2 Cardiomyocyte apoptosis and autophagy

Some lncRNAs have been identified to be correlated with cardiomyocyte apoptosis and autophagy during the process of diabetic cardiomyopathy.^65^ Recently, our research group found that myocardial infarction–associated transcript (MIAT) was upregulated in the diabetic myocardium, while MIAT knockdown could reduce cardiomyocyte apoptosis and improve cardiac dysfunction.^66^ We then further investigated the molecular mechanisms involved and found that MIAT acted as a ceRNA to increase DAPK2 expression by sponging miR‐22‐3p, thus leading to elevated cardiomyocyte apoptosis.^66^ Moreover, we generated a diabetic rat model induced by streptozocin and found that metastasis‐associated lung adenocarcinoma transcript 1 (MALAT1) expression was increased in the diabetic heart. MALAT1 knockdown was associated with improved cardiac function, partly through the suppression of cardiomyocyte apoptosis.^67^

The lncRNA‐H19 is a member of conserved imprinted gene family and participates in embryonic development and growth regulation. In the previous study, we investigated the pathological roles of H19 in the development of diabetic cardiomyopathy. The results indicated that H19 was downregulated in the diabetic myocardium and high glucose treatment contributed to cardiomyocyte apoptosis by modulating H19/miR‐675/VDAC1 pathway.^68^ In addition, another study by Zhuo et al^69^ revealed that high glucose could downregulate H19 expression and promote autophagy in myocardial cells. H19 overexpression could reduce DIRAS3 expression, increase mTOR phosphorylation and suppress autophagy activation. Thus, H19 is involved in the modulation of autophagy in diabetic cardiomyopathy by epigenetically silencing of DIRAS3.

### 3.3 Inflammation

Inflammation has a significant involvement in the progression of diabetic cardiomyopathy.^65^ Our research group previously found that MALAT1 expression was increased in the diabetic heart, and its knockdown could improve cardiac systolic function and reduce the levels of inflammatory cytokines such as TNF‐α, IL‐6 and IL‐1β in the diabetic myocardium, thus indicating that MALAT1 might be related to the inflammatory response in diabetic cardiomyopathy.^70^ Moreover, another study by Puthanveetil et al^71^ reported that MALAT1 upregulated inflammatory mediators TNF‐α and IL‐6 in endothelial cells treated with high glucose through activation of serum amyloid antigen 3.

In previous studies, our research group generated a rat model of diabetic cardiomyopathy and conducted a microarray to determine the differentially expressed lncRNAs in cardiac tissue. We then investigated the pathological effects of MIAT, MALAT1 and H19 in the development of diabetic cardiomyopathy, mainly focusing on the mechanisms of apoptosis and inflammation.^66^, ^67^, ^68^, ^70^ However, it remains a challenge to translate basic research results into clinical practice. Recently, it has been documented that circulating lncRNAs such as LIPCAR, SENCR and MIAT are valuable predictors of left ventricular diastolic function and remodelling in diabetic patients.^72^

## 4 circRNAs

circRNAs are produced from precursor mRNAs by the back‐splicing of exons in eukaryotes and are widely expressed in a tissue‐specific and developmental stage–specific pattern.^73^ circRNAs differ from linear RNAs in that they are circular molecules with covalently closed loop structures and lack 5′‐3′ polarity or a polyadenylated tail. circRNAs may function as miRNA sponges to inhibit the translation of mRNAs, which is the most common regulatory mechanism. In addition, circRNAs can alter gene expression by regulating splicing or transcription and by interacting with RNA‐binding proteins.^74^

The development of cardiac fibrosis is a key event in the pathogenesis of diabetic cardiomyopathy. Tang et al^75^ revealed that circRNA_000203 was upregulated in diabetic myocardium and was correlated with increased expression of α‐SMA, Col1a2 and Col3a1 in cardiac fibroblasts. Moreover, circRNA_000203 could sponge miR‐26b‐5p to derepress the downstream targets of Col1a2 and CTGF, which contributes to the expression of fibrosis‐associated genes in cardiac fibroblasts. Similarly, Zhou et al^76^ showed the involvement of another circRNA in the regulation of diabetic myocardial fibrosis. They found that circRNA_010567 modulated miR‐141 and its target gene TGF‐β1 and mediated fibrosis‐associated protein resection. Thus, circRNA_010567/miR‐141/TGF‐β1 pathway plays a critical regulatory role in myocardial fibrosis, promoting the development of diabetic cardiomyopathy.

circHIPK3 is a particularly abundant circRNA involved in the metabolic dysregulation and tumorigenesis.^77^, ^78^, ^79^ Combined with circRNA screening and functional confirmation, our research group suggested that circHIPK3 was significantly upregulated in the diabetic myocardium, and circHIPK3 might act as a ceRNA to increase VAMP7 expression by sponging miR‐143‐5p, which resulted in elevated cardiomyocyte autophagy and contributed to the development of diabetic cardiomyopathy (unpublished data).

Compared with miRNAs and lncRNAs, the understanding of circRNAs in the molecular mechanisms of diabetic cardiomyopathy is still limited. circRNAs can modulate gene expression by functioning as RNA‐binding protein sequestering agents, nuclear transcriptional regulators and miRNA sponges.^74^ Emerging evidence has shown that circRNAs play crucial roles in multiple cardiovascular diseases and may serve as useful biomarkers due to their abundance and stability.^80^ Future research will focus on two directions. Firstly, to further investigate the pathological roles of circRNAs in the development of diabetic cardiomyopathy. Secondly, to identify more circulating circRNAs as biomarkers for the diagnosis and prognosis of diabetic cardiomyopathy.

## 5 CONCLUSION

**Figure 2:**
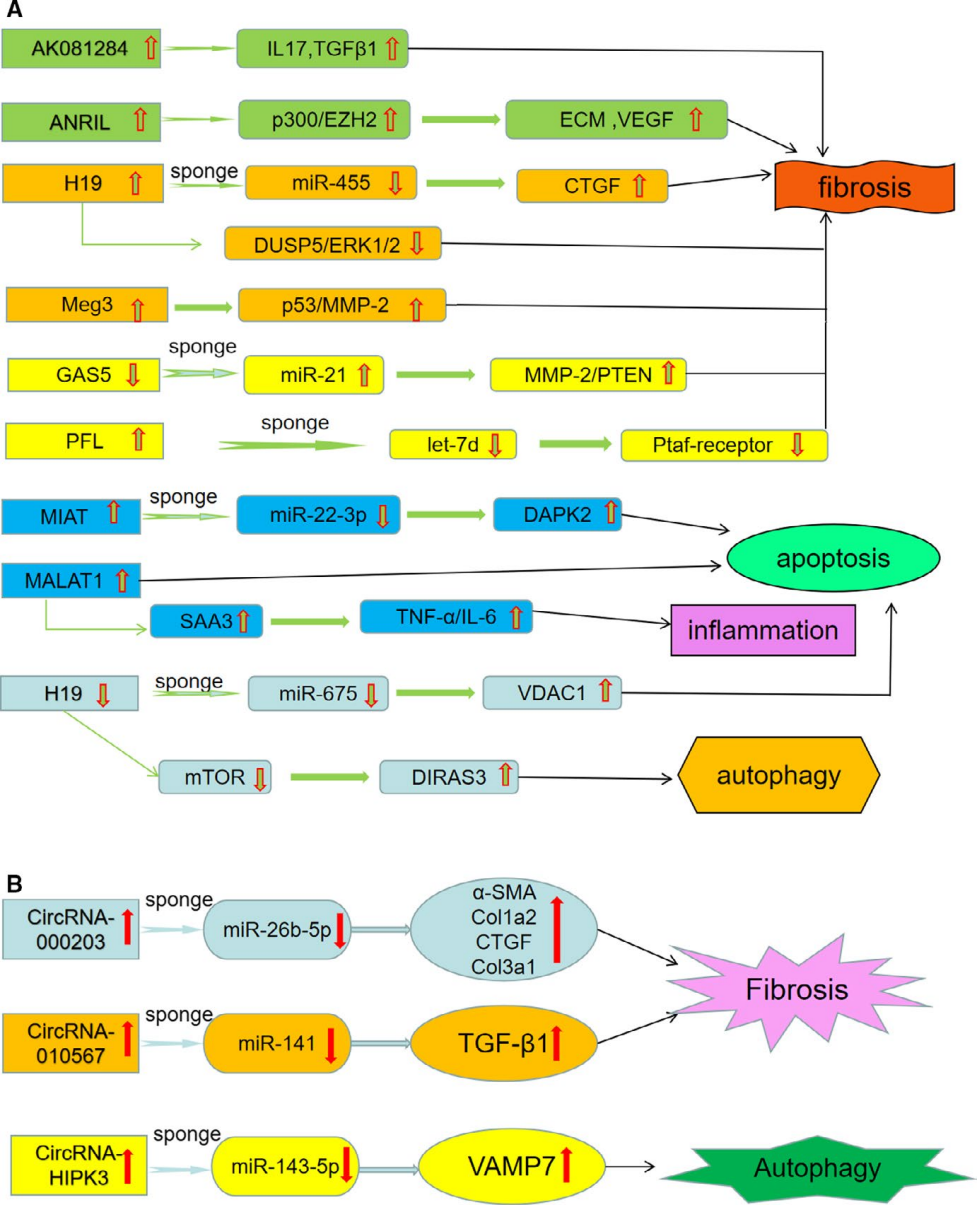
A, Involvement of lncRNA in the pathogenesis of diabetic cardiomyopathy; B, involvement of circRNA in the pathogenesis of diabetic cardiomyopathy

In the present review, we summarize the recent progress in the involvement of ncRNAs in the pathogenesis of diabetic cardiomyopathy. As shown in Table 1, we present a variety of miRNAs responsible for the regulation of cardiomyocyte hypertrophy, apoptosis and autophagy, myocardial fibrosis, oxidative stress and inflammatory response, which are important mechanisms associated with diabetic cardiomyopathy. Unlike miRNAs, there are fewer studies on lncRNAs and circRNAs involved in the molecular mechanisms of diabetic cardiomyopathy (Figure 2). In our previous studies, several ncRNAs including MIAT, MALAT1, H19 and circHIPK3 have been suggested to participate in the modulation of cardiomyocyte apoptosis and autophagy and consequently result in the development of diabetic cardiomyopathy.

**Table 1:** The role of miRNA in the pathogenesis of diabetic cardiomyopathy

miRNAs	Expression	Target genes	Pathological mechanism	Reference
miR‐1	Downregulated	Mef2a/Gata4	Anti‐hypertrophy	16
	Upregulated	IGF‐1	Pro‐apoptosis	25
	Downregulated	RyR2	Anti‐oxidative stress	41
miR‐30c/181a	Downregulated	p53/p21	Anti‐hypertrophy/anti‐apoptosis	18
miR‐133a	Downregulated	SGK1/IGF1R	Anti‐hypertrophy	19
	Downregulated	CTGF/TGF‐β1/ FGF1	Anti‐fibrosis/DNA methylation	10, 13
miR‐150	Downregulated	p300	Anti‐hypertrophy/anti‐oxidative stress	9
miR‐373	Downregulated	MEF2C	Anti‐hypertrophy/anti‐oxidative stress	20
miR‐208a	Upregulated	Myostatin/GATA4	Pro‐hypertrophy	21
	Upregulated	pim‐1	Pro‐apoptosis	28
miR‐451	Upregulated	CAB39	Pro‐hypertrophy	22
miR‐195	Upregulated	BCL‐2/Sirt1	Pro‐apoptosis/pro‐hypertrophy/pro‐oxidative stress	23
miR‐30b	Upregulated	Bcl2	Pro‐apoptosis	26
miR‐206	Upregulated	Hsp60	Pro‐apoptosis	27
miR‐144	Upregulated	Nrf2	Pro‐apoptosis/pro‐oxidative stress	11
miR‐320	Upregulated	VEGF‐c/Flk‐1/IGF‐1/IGF‐1R/FGFs	Pro‐apoptosis	29
miR‐378	Upregulated	IGFR1	Pro‐apoptosis	30
miR483‐3p	Upregulated	IGF1	Pro‐apoptosis	31
miR‐34a	Upregulated	Bcl‐2	Pro‐apoptosis	32
miR‐221	Upregulated	p27	Impaired autophagy	34
miR‐30c	Upregulated	BECN1	Inhibited autophagy	33
miR‐9	Downregulated	ELAVL1	Anti‐pyroptosis	36
miR‐30d	Upregulated	foxo3a	Pro‐pyroptosis	15
miR‐15a/b	Downregulated	TGFaR1/CTGF	Anti‐fibrosis	37
miR‐21	Upregulated	DUSP8	Pro‐fibrosis	38
miR‐29	Downregulated	COL1A1/1A2/3A1	Anti‐fibrosis	39
	Upregulated	MCL‐1	Cardiac structural damage	46
miR‐200b	Downregulated	VEGF/p300	Anti‐fibrosis	40
	Upregulated	ZEB1/2	Pro‐inflammation	48
miR‐22	Downregulated	Sirt1	Anti‐oxidative stress	42
miR‐200c	Upregulated	COX‐2/ZEB1/2	Pro‐oxidative stress/pro‐inflammation	43, 48
miR‐503	Upregulated	CPDT	Pro‐oxidative stress	44
miR‐141	Upregulated	Slc25a3	Mitochondrial dysfunction	47
miR‐146a	Downregulated	IL6, TNFα, IL‐1β, MCP‐1, NF‐κB, Col1α1, Col4α1	Anti‐inflammation	49
miR‐301a	Upregulated	Kv4.2	Electrical remodelling	14
miR193‐5p	Upregulated	IGF2	Pro‐angiogenesis	50

The available evidence indicates that ncRNAs mainly function as ceRNAs to modulate the expression of target genes by sponging miRNAs. However, there are several other mechanisms such as transcriptional modulation, post‐transcriptional processing and interaction with RNA‐binding proteins. In the future work, we should further clarify the molecular mechanisms of ncRNAs in the development of diabetic cardiomyopathy. Compared with miRNAs, current studies on lncRNAs and circRNAs in diabetic cardiomyopathy are relatively fewer because they were discovered later. Therefore, it is urgent for us to elucidate the pathological mechanisms of lncRNAs and circRNAs in diabetic cardiovascular complications.

Recently, cumulative evidence has demonstrated that circulating ncRNAs are potential biomarkers for the diagnosis and prognosis of various cardiovascular diseases. In the future, more clinical research should be conducted to evaluate the diagnostic and prognostic value of circulating ncRNAs in diabetic cardiomyopathy. In addition, some ncRNAs are emerging as novel therapeutic targets in the treatment of diabetic cardiovascular complications. RNA interference drugs and antisense oligonucleotides are well‐known molecular tools for regulating gene expression through sequence‐specific interactions with RNA. Furthermore, recent research evidence suggests that CRISPR genome editing technology is able to effectively modify the expression of ncRNAs and has a broad application prospect in the treatment of diabetic cardiomyopathy.

## CONFLICTS OF INTEREST

The authors declare that there are no conflicts of interest.

## AUTHOR CONTRIBUTIONS

Wei Zhang and Weiting Xu wrote the manuscript; Yu Feng and Xiang Zhou revised the manuscript.

## Data Availability

I confirm that I have included a citation for available data in my references section.
